# Supplementary figures and images for: Multi-omic analysis reveals nitric oxide dependent remodeling in classically activated macrophages and identifies negative regulation mediated by AKR1A1
Source: Redox Biol. 2026 Apr 22;93:104181. doi: 10.1016/j.redox.2026.104181 (PMC13137910; doi:10.1016/j.redox.2026.104181)

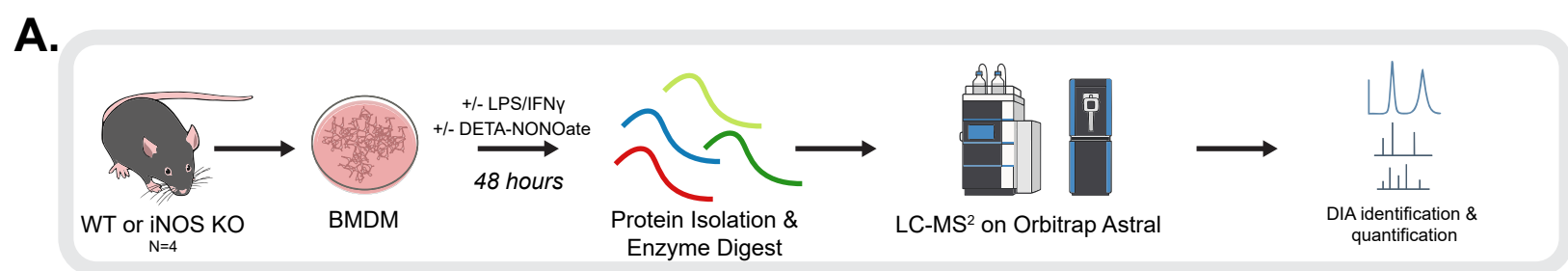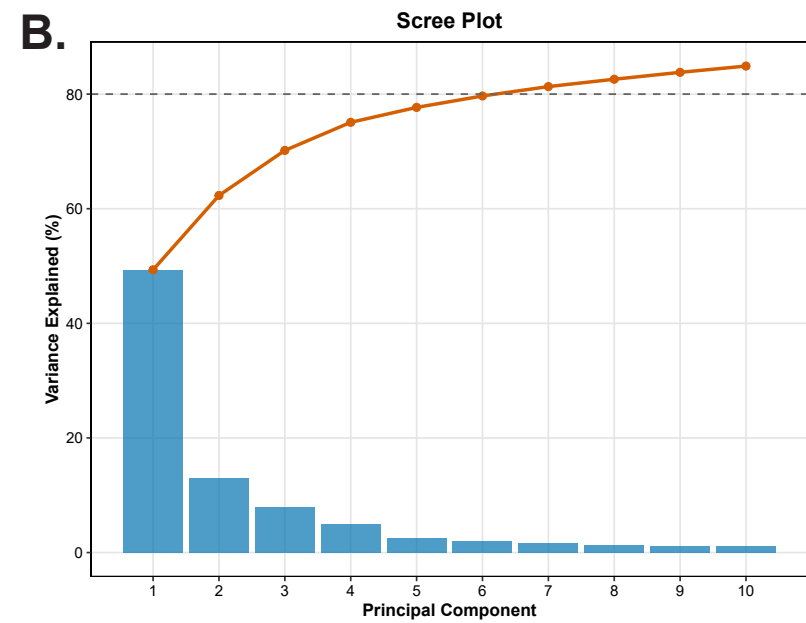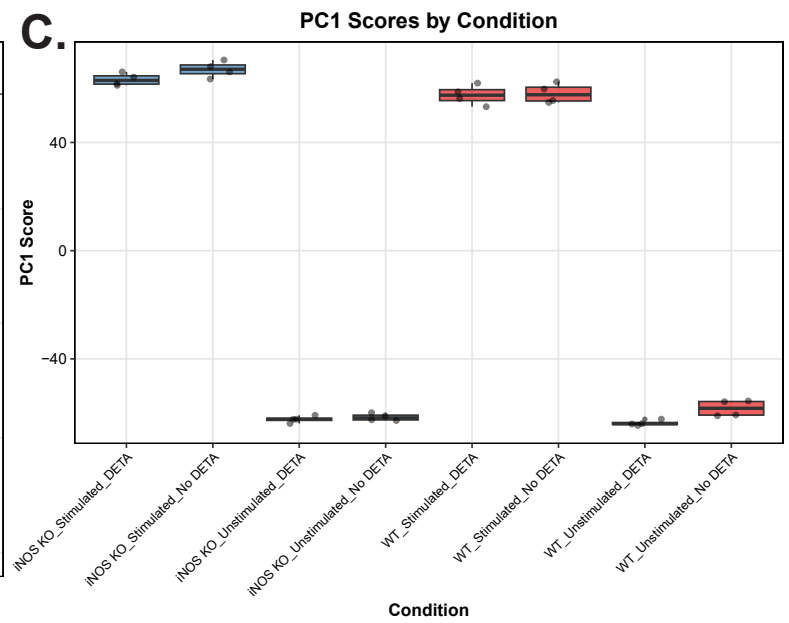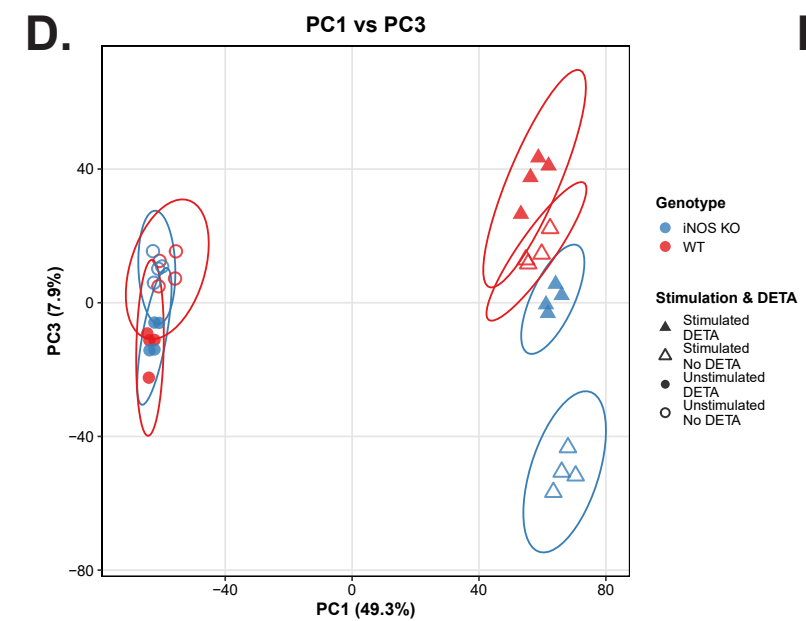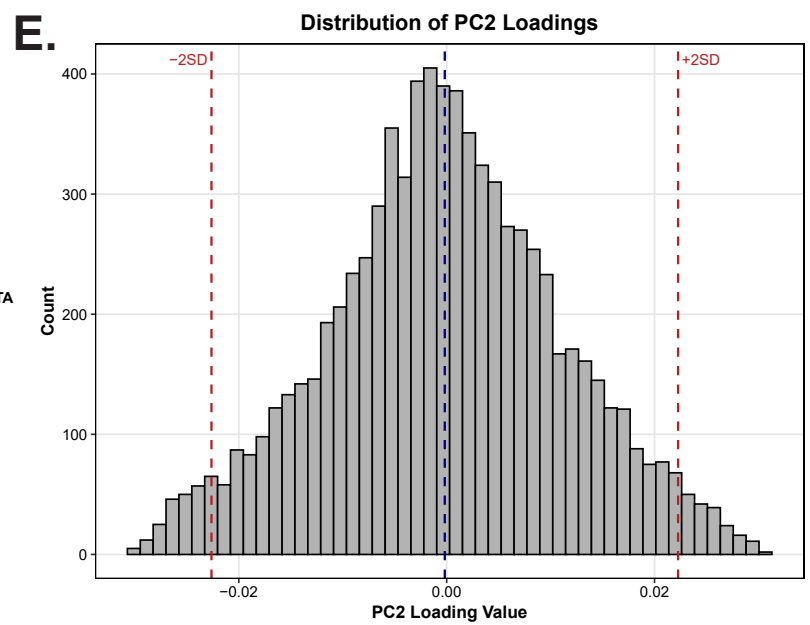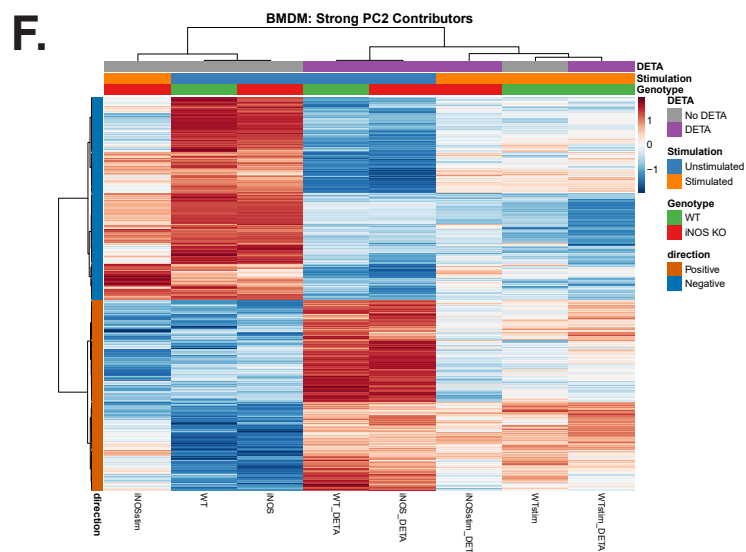

Supplement: Supplementary file 1 — Supplemental Figure 1Principal component analysis (PCA) of BMDM proteomic data reveals NO•-dependent protein signatures (A) Experimental design and proteomic workflow for BMDMs from wild-type (WT) or iNOS knockout (iNOS KO) mice that were unstimulated or stimulated with LPS/IFNγ for 48-h with or without DETA-NONOate treatment, n = 4 biological replicates per genotype. (B–F) Principal component analysis (PCA) was performed on log2-transformed protein intensities from BMDM cultures across all experimental conditions (WT and iNOS KO, unstimulated and stimulated with LPS/IFNγ for 48 h, ± DETA-NONOate). (B) Scree plot showing variance explained by the first 10 principal components. (C) Distribution of PC1 scores across experimental conditions. Box plots show median, interquartile range (box), 1.5x interquartile range (whiskers), and individual replicates (points). (D) PCA map along PC1 and PC3. Ellipses represent 95% confidence intervals for each of the 8 unique condition groups. (E) Distribution of PC2 loadings across all proteins (n = 7937). Proteins with loadings >2 standard deviations from the mean were classified as strong PC2 contributors (n = 209 positive contributors; n = 223 negative contributors). Dashed vertical lines indicate ±2SD thresholds. (F) Heatmap showing Z-score of top positive or negative PC2 loading proteins across conditions (n = 432). Hierarchical clustering based on Pearson correlation distance (Ward.D2 linkage). Color scale: blue (low) and red (high) expression relative to mean.Multimedia Component 1 [file mmc1.pdf]

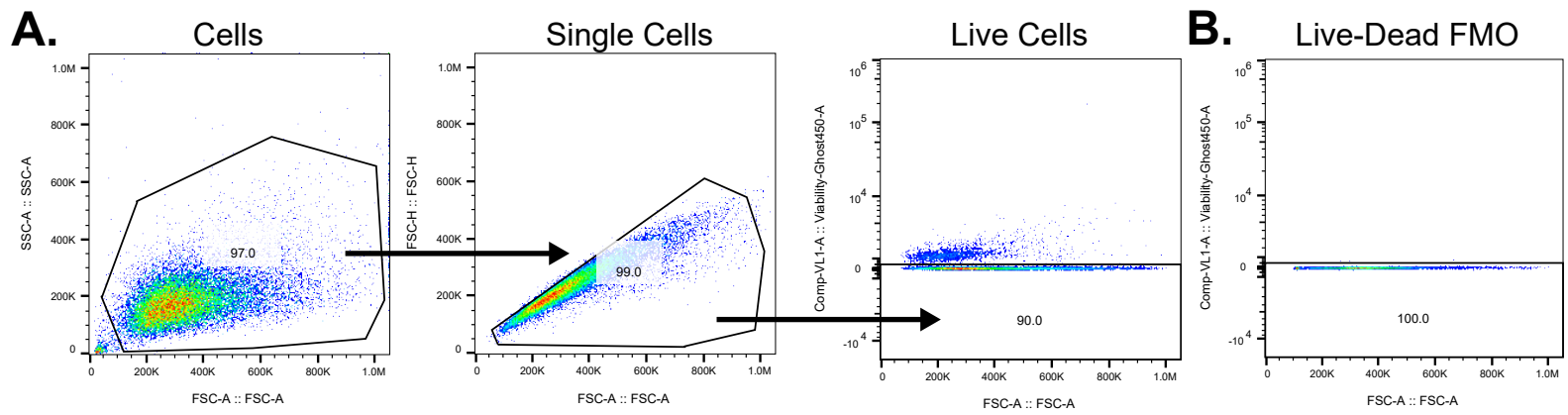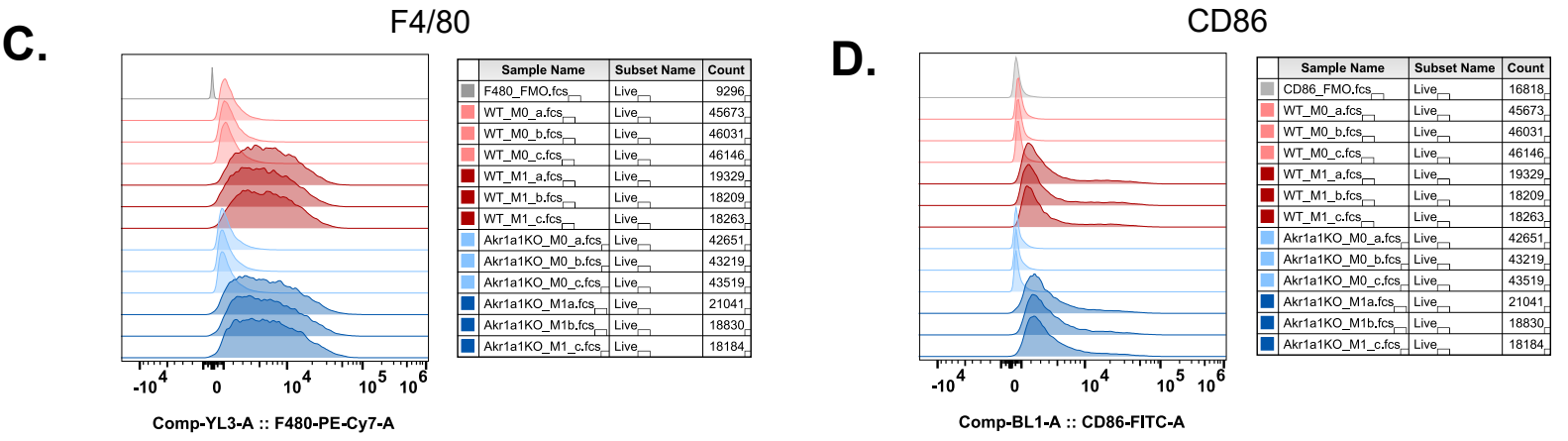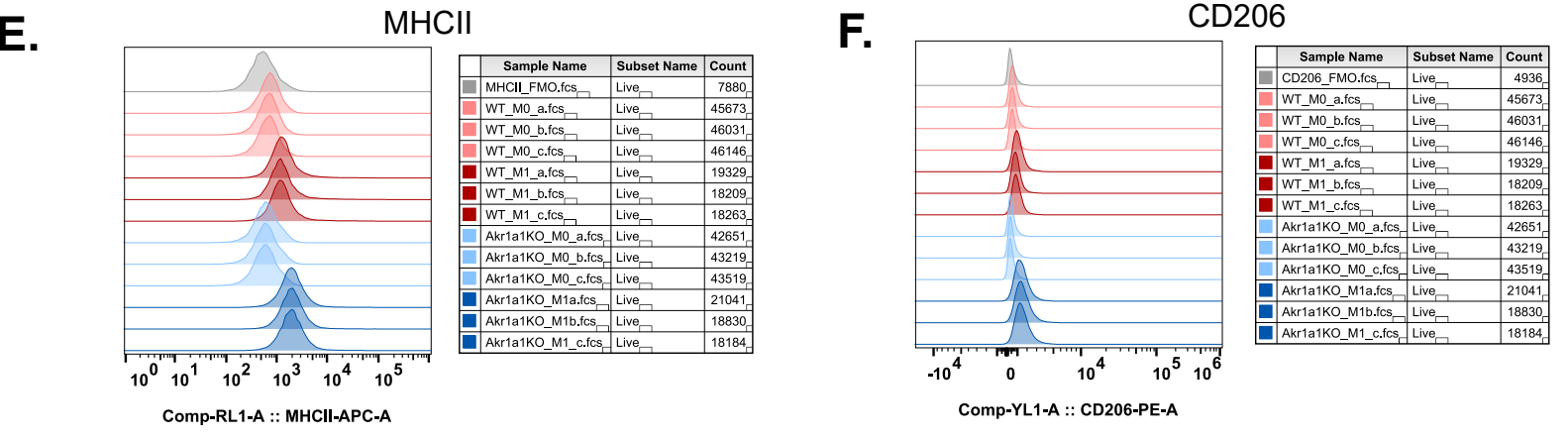

Supplement: Supplementary file 2 — Supplemental Figure 10Gating Strategy and Flow Cytometry Histograms (A) Representative gating strategy for flow cytometry. (B) Viability Ghost450 (Live-Dead stain) FMO sample with ‘Live Cells’ gating.(C–F) Flow cytometry histograms (WT RAW264.7 cells in red: unstimulated: light red, stimulated: dark red; Akr1a1 KO RAW264.7 cells in blue: unstimulated: light blue, stimulated: dark blue) in triplicate and corresponding FMO (grey) for each fluorophore-conjugated antibody: (C) F480-PE-Cy7, (D) CD86-FITC, (E) MHCII-APC, and (F) CD206-PE.Multimedia Component 10 [file mmc10.pdf]

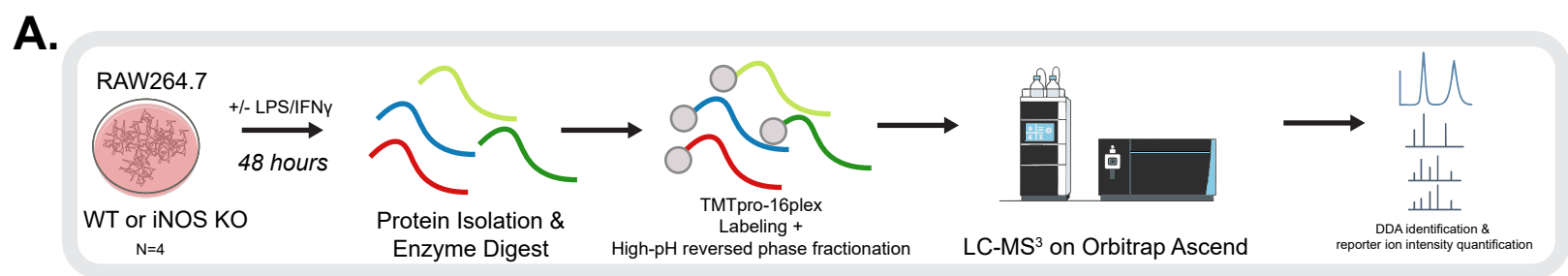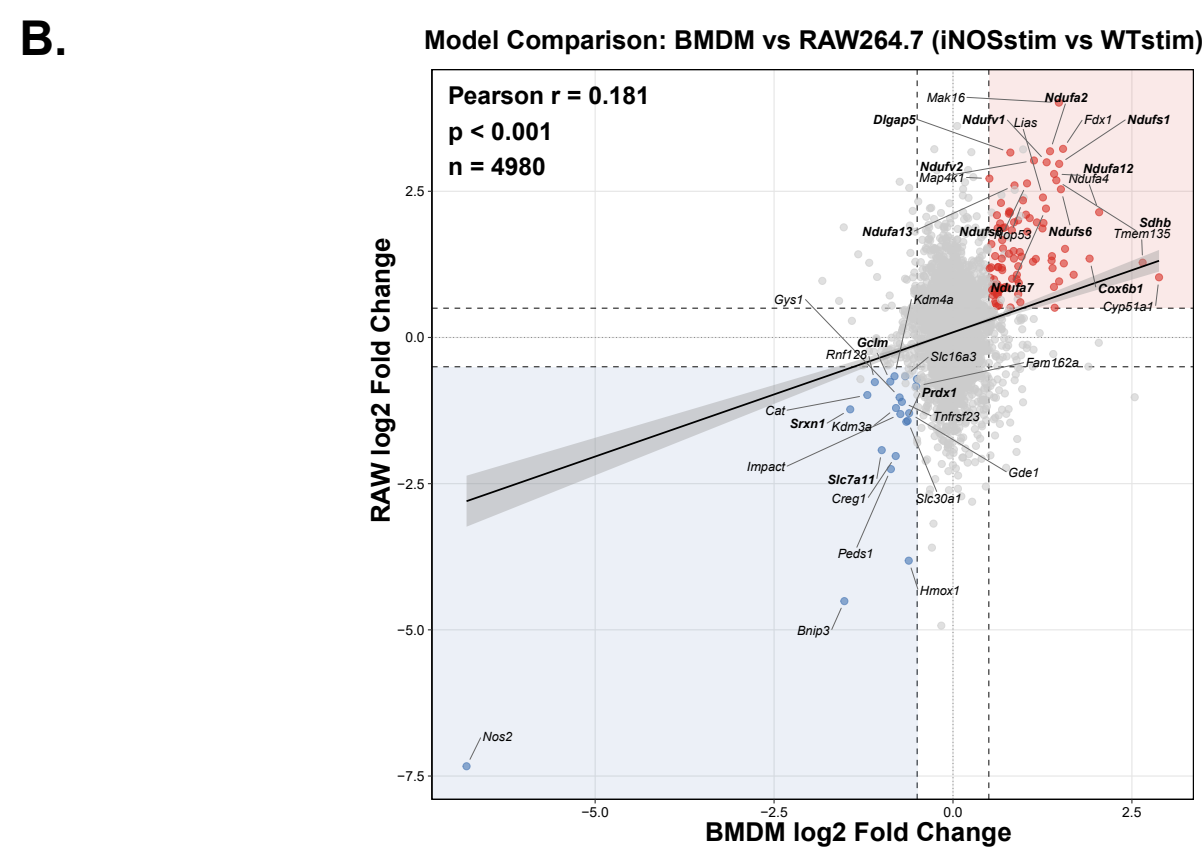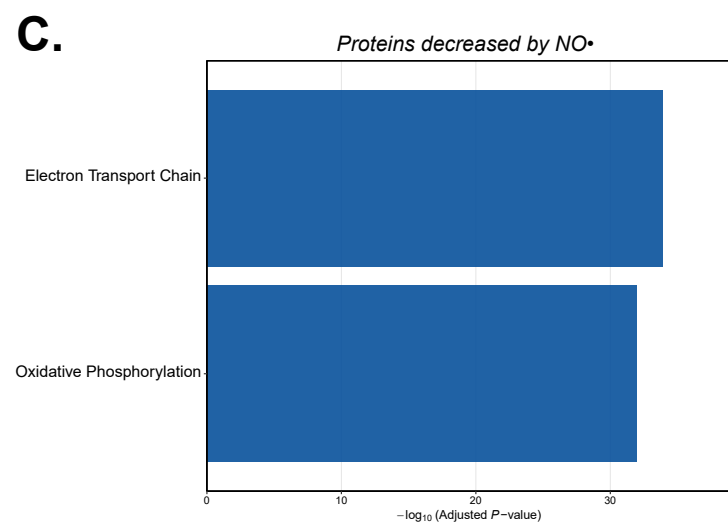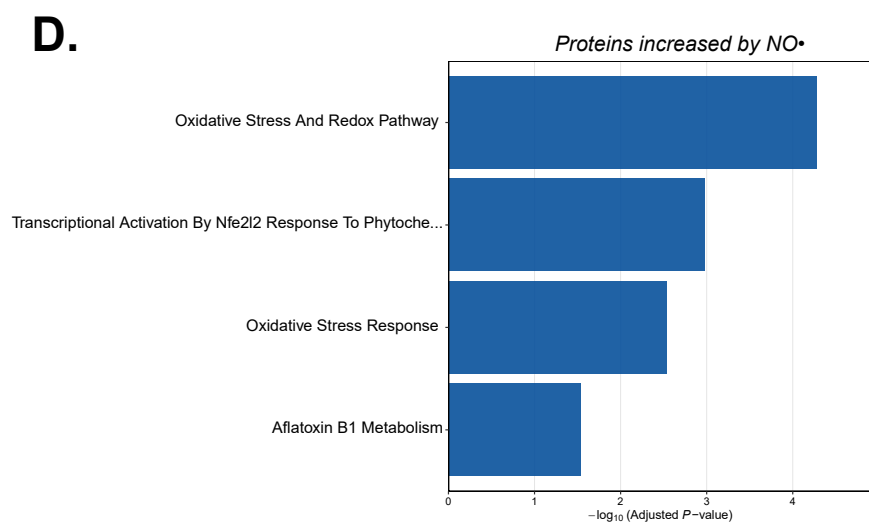

Supplement: Supplementary file 3 — Supplemental Figure 2NO-dependent proteomic changes across cell models (A) Experimental design and proteomic workflow for RAW264.7 macrophage-like cells. Wildtype (WT) or iNOS knockout (KO) RAW264.7 cells were stimulated with or without LPS/IFNγ for 48 h. TMTpro 16-plex labeling with 4 biological replicates per condition. (B) Cross-model correlation analysis comparing iNOS-dependent proteomic changes (log2 fold change: stimulated iNOS KO versus stimulated WT) between BMDM and RAW264.7 datasets for overlapping proteins. Dashed lines indicate |log2 fold change| = 0.5 threshold. Shaded regions highlight concordant quadrants. Points are colored by significance: both models significant (p-adj <0.05) and concordantly increased (red) or decreased (blue) with |log2FC| > 0.5 in both datasets. Pearson correlation with linear regression shown (95% CI as shaded band). (C–D) Pathway enrichment analysis (Mouse WikiPathway 2024 via Enrichr) for concordantly decreased (C) or increased (D) proteins from panel B.Multimedia Component 2 [file mmc2.pdf]

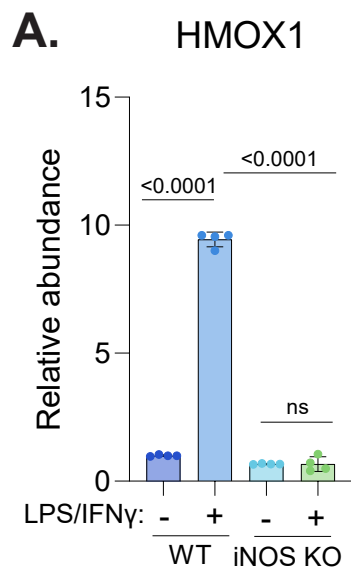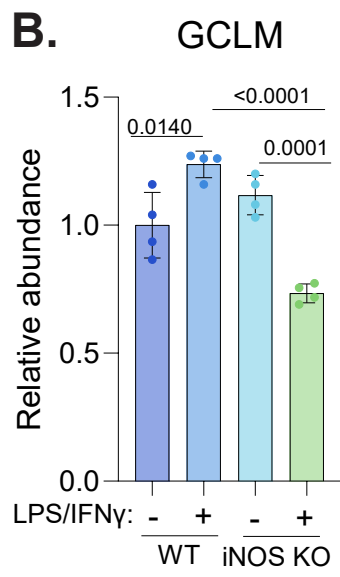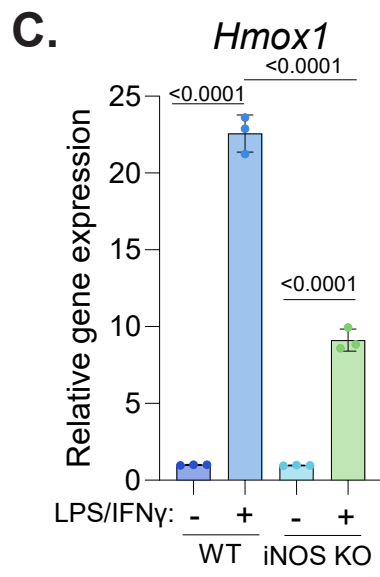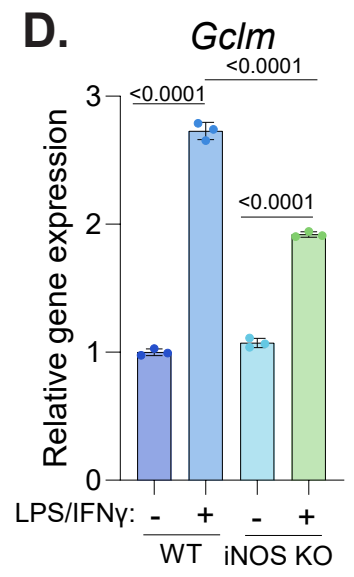

Supplement: Supplementary file 5 — Supplemental Figure 4Protein and transcript levels of classic NRF2 targets (A-B) Relative abundance of (A) HMOX1 and (B) GCLM in wildtype (WT) or iNOS knockout (KO) RAW264.7 macrophage-like cells that are unstimulated or stimulated with LPS/IFNγ for 48 h. Data represents the mean ± standard deviation (SD), n = 4 biological replicates per condition. (C–D) Relative gene expression of (C) Hmox1 and (D) Gclm generated from the normalized gene counts in transcriptomic dataset from wildtype (WT) or iNOS knockout (KO) RAW264.7 cells that are unstimulated or stimulated with LPS/IFNγ for 48 h. Data represents the mean ± SD, n = 3 biological replicates per genotype per condition. Statistical comparisons were performed using unpaired two-tailed t-test with p-value reported.Multimedia Component 4 [file mmc4.pdf]

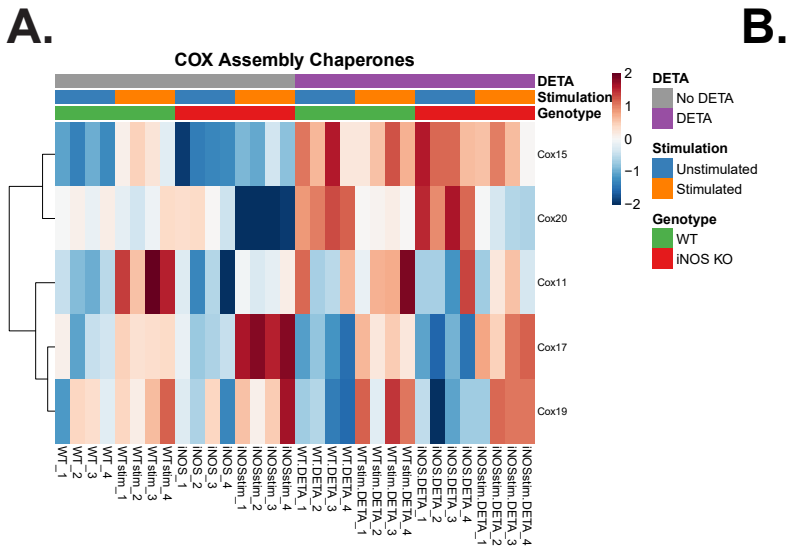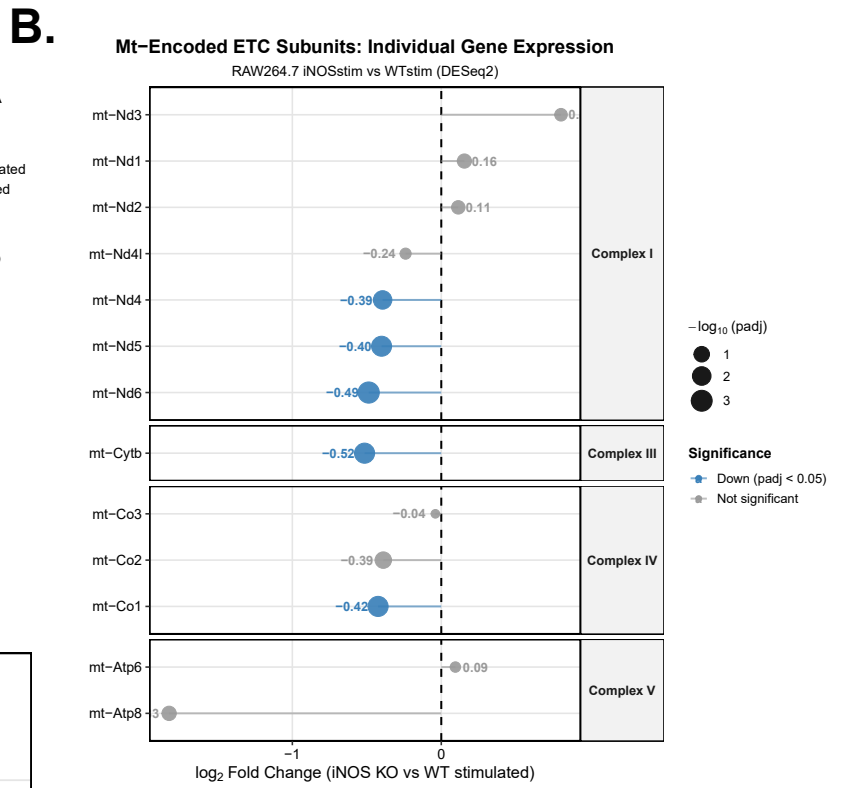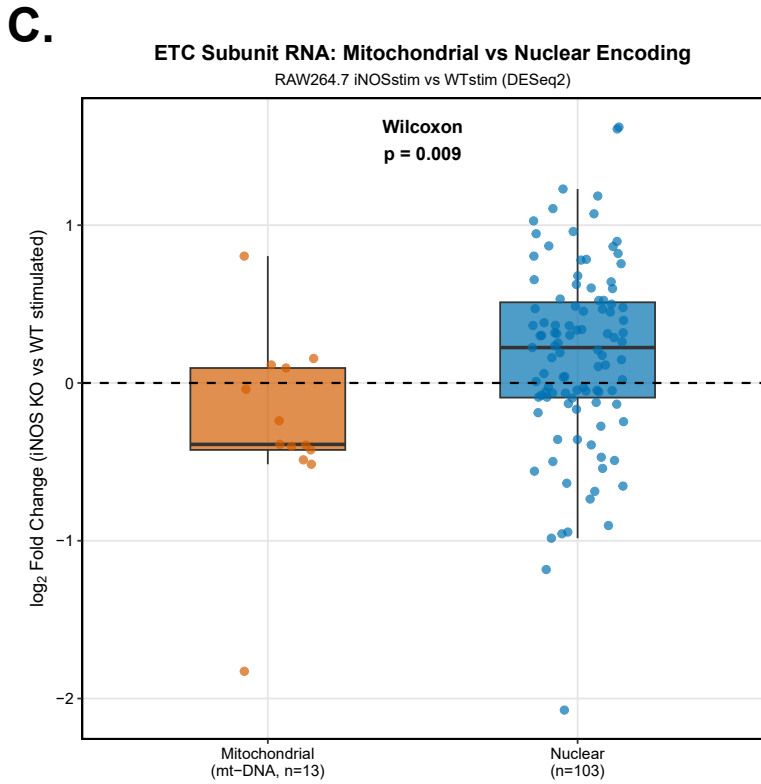

Supplement: Supplementary file 6 — Supplemental Figure 5Changes in ETC components in response to iNOS (A) Heatmap showing changes in COX Assembly Chaperone proteins (Cox10, Cox11, Cox15, Cox17, Cox19, Cox20) in BMDM across eight experimental conditions: unstimulated (unstim) or stimulated (stim) with LPS/IFNγ for 48 h in WT or iNOS KO genotypes; ± DETA-NONOate (DETA). Colors represent row-wise Z-score normalized protein abundance (blue = decreased, red = increased relative to row mean). Rows (proteins) clustered by Pearson correlation; columns ordered by experimental condition. n = 4 biological replicates per condition (individual replicates designated by "_1″ through "_4″ suffix). (B) Mitochondrial encoded ETC subunits subset from transcriptomic dataset described in Supplemental Fig. 3A. Colored by significance: blue indicates p-adj <0.05, grey indicates not significant. Dot size corresponds to -log10(p-adj) value. (C) Boxplots are grouped by mitochondrial (orange) versus nuclear (orange) encoded subset from transcriptomic datasets described in Supplemental Fig. 3A. Statistical comparison using non-parametric Wilcoxon Rank-Sum test with p-value reported.Multimedia Component 5 [file mmc5.pdf]

**A.**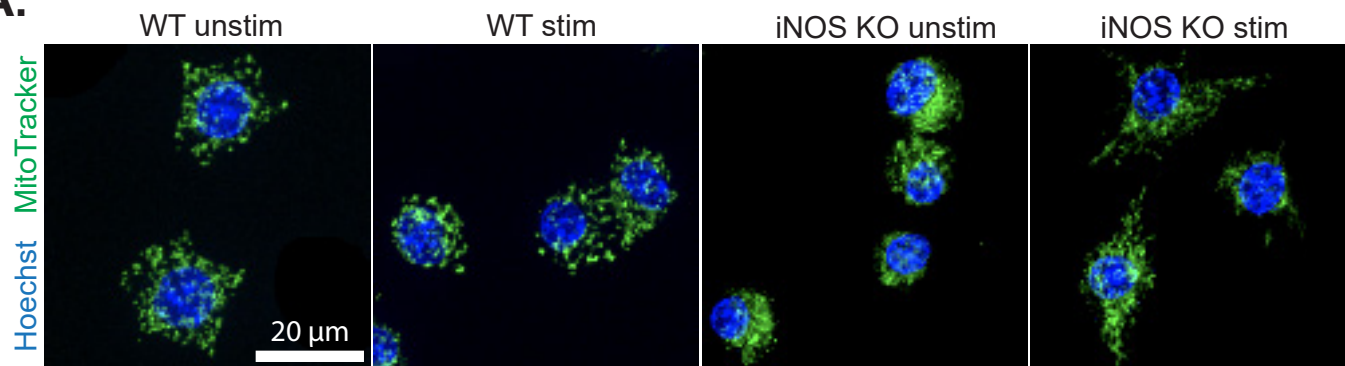**B.**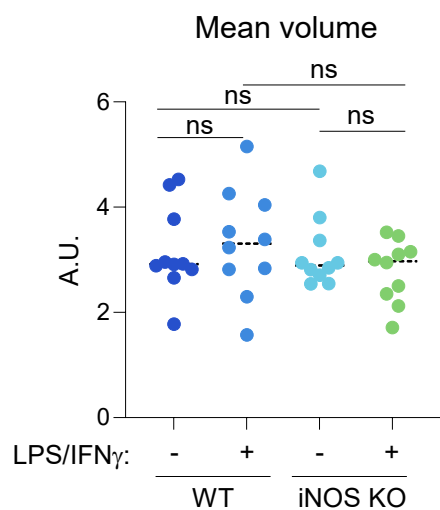**C.**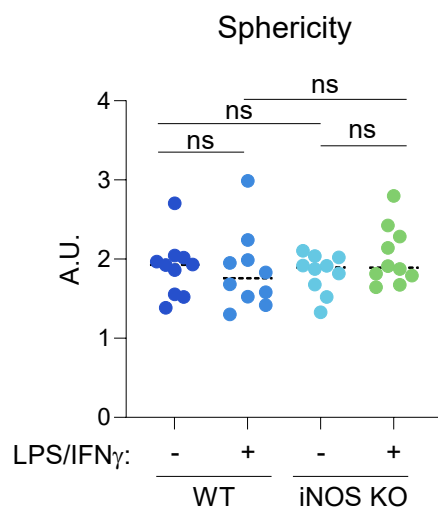**D.**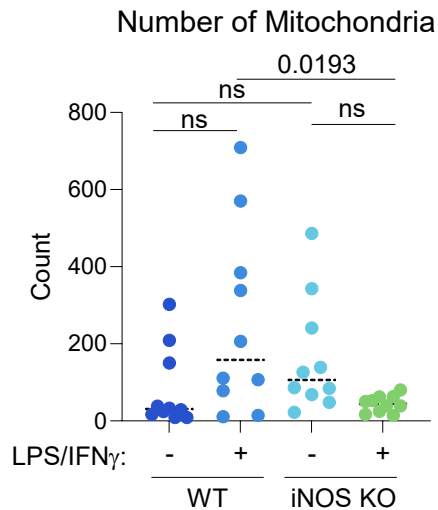**E.**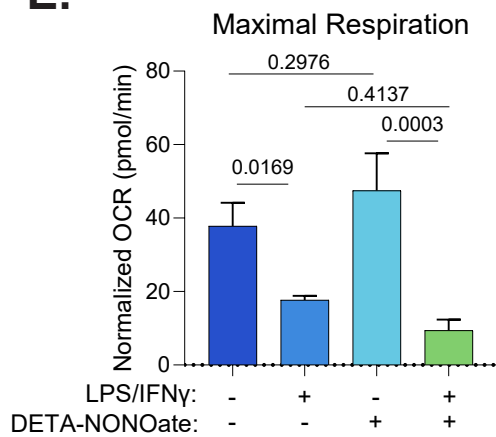

Supplement: Supplementary file 7 — Supplemental Figure 6Mitochondrial morphology quantified in stimulated or unstimulated WT and iNOS KO RAW264.7 cells (A) Representative live cell confocal images of wildtype (WT) and iNOS knockout (KO) RAW264.7 cells with or without LPS/IFNγ stimulation for 24 h. Nucleus (blue, Hoechst), mitochondria (green, MitoTracker Green). Scale bar = 20 μm. (B–D) Quantification of mean mitochondrial volume (B), sphericity (C), and number per cell (D) from MitoTracker-labeled cells analyzed with MitoAnalyzer. Data represent mean ± standard deviation (SD); n = 10 cells from 2 independent experiments. (E) Normalized maximal respiration rate, measured by oxygen consumption after cells are treated with uncouple agent FCCP (0.5 μM), for in BMDMs that are unstimulated or stimulated with LPS/IFNγ ± 200 μM DETA-NONOate for 48-h. Data represent mean ± SD, n = 3 biological replicates. Statistical comparisons by one-way ANOVA with Tukey's post hoc test for multiple comparisons with p-value reported; ns indicates not significant.Multimedia Component 6 [file mmc6.pdf]

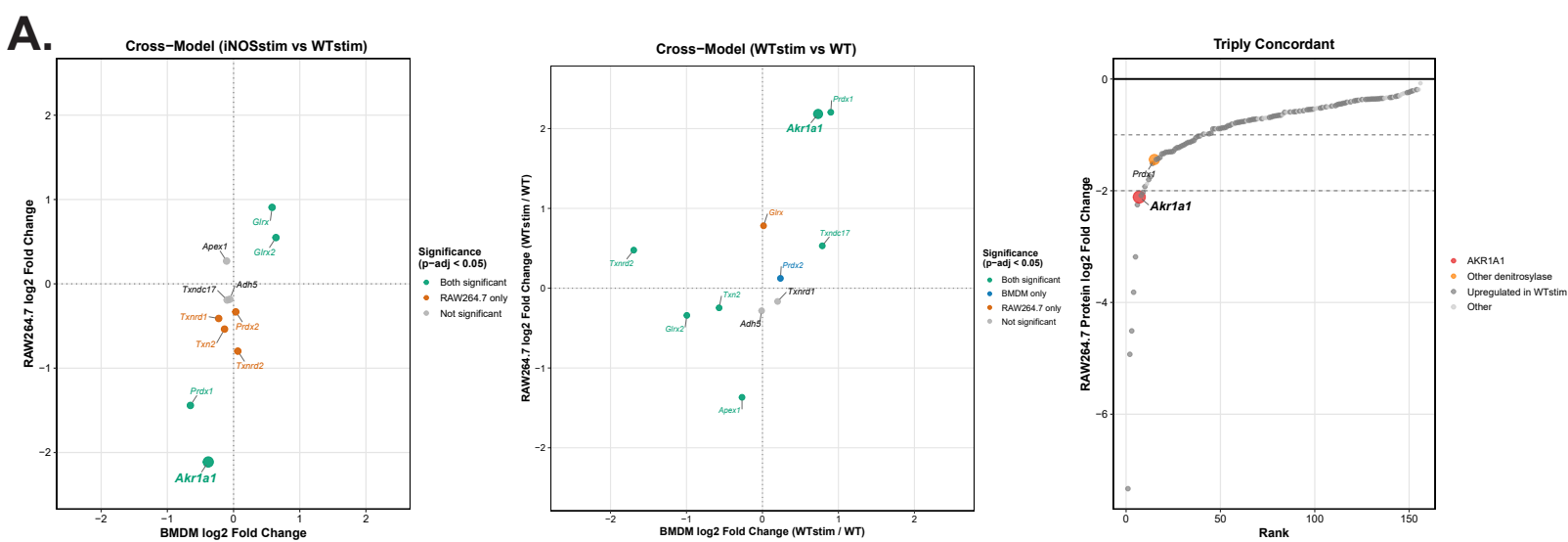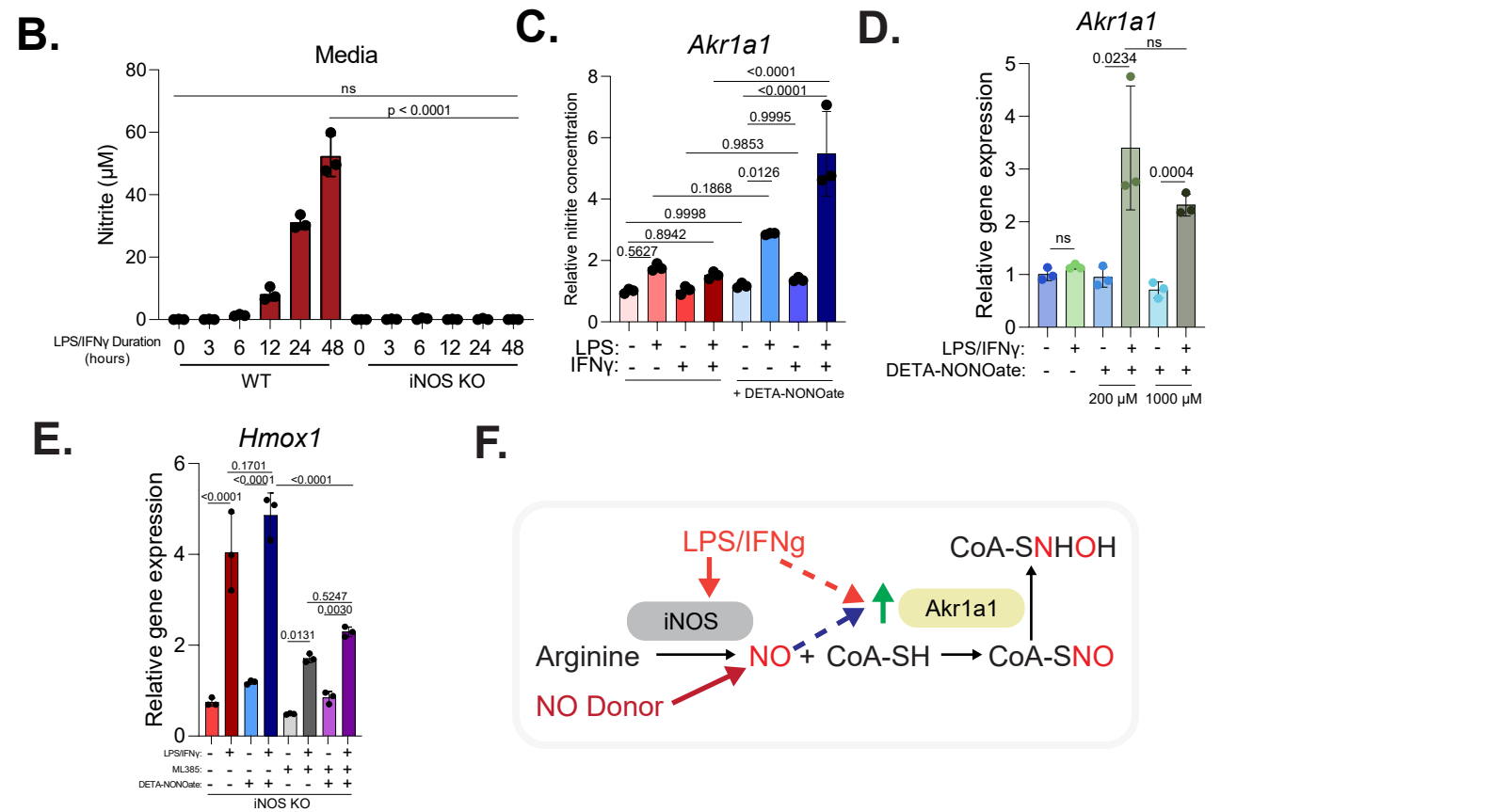

Supplement: Supplementary file 8 — Supplemental Figure 7The upregulation of AKR1A1 in response to stimulation and NO (A) Regulation of AKR1A1 compared to other proteins involved in nitrosylation regulation; left: log2-fold changes in proteins involved in de-nitrosylation by iNOS knockout in stimulated RAW264.7 and BMDM; middle: log2-fold changes in protein level induced by stimulation in wildtype RAW264.7 and BMDM. Points are colored by significance: changes that are significant (p-adj <0.05) in both cell models (green), in BMDM only (blue), in RAW264.7 cells only (orange), or not significant in either models (grey); right: plot as described in Fig. 4A but subset with proteins involved in de-nitrosylation (Akr1a1 colored red, others on this list colored orange). Dark grey highlights proteins significantly induced by LPS/IFNγ in WT cells (p-adj <0.05, log2FC > 0). (B) Nitrite accumulation in culture media from cells in (Fig. 4B) measured by Griess assay. Data represents mean ± standard deviation (SD), n = 3 biological replicates. (C) Relative mRNA expression of Akr1a1 in iNOS KO BMDMs treated with all combinations of LPS, IFNγ, and DETA-NONOate (200 μM) for 48 h. Expression normalized to Hnrpab reference gene using ΔΔCt method. Data represents mean ± SD, n = 3 biological replicates. (D) Relative mRNA expression of Akr1a1 in iNOS KO BMDMs stimulated with or without stimulation of LPS/IFNγ ± 200 μM or 1000 μM DETA-NONOate for 48 h. Expression normalized to Hnrpab. Data represents mean ± SD, n = 3 biological replicates. (E) Relative mRNA expression of Hmox1, a canonical Nrf2 target, in iNOS KO BMDMs unstimulated or stimulated with LPS/IFNγ ± DETA-NONOate ± ML385 (Nrf2 inhibitor, 10 μM) for 48 h. Expression normalized to Hnrpab. Data represents mean ± SD, n = 3 biological replicates. (F) Schematic depicting co-regulation of Akr1a1 by classical activation signals and NO. Statistics: For B-E, statistical comparisons were performed using one-way ANOVA with Tukey's post hoc test for multiple comparisons with [file mmc7.pdf]

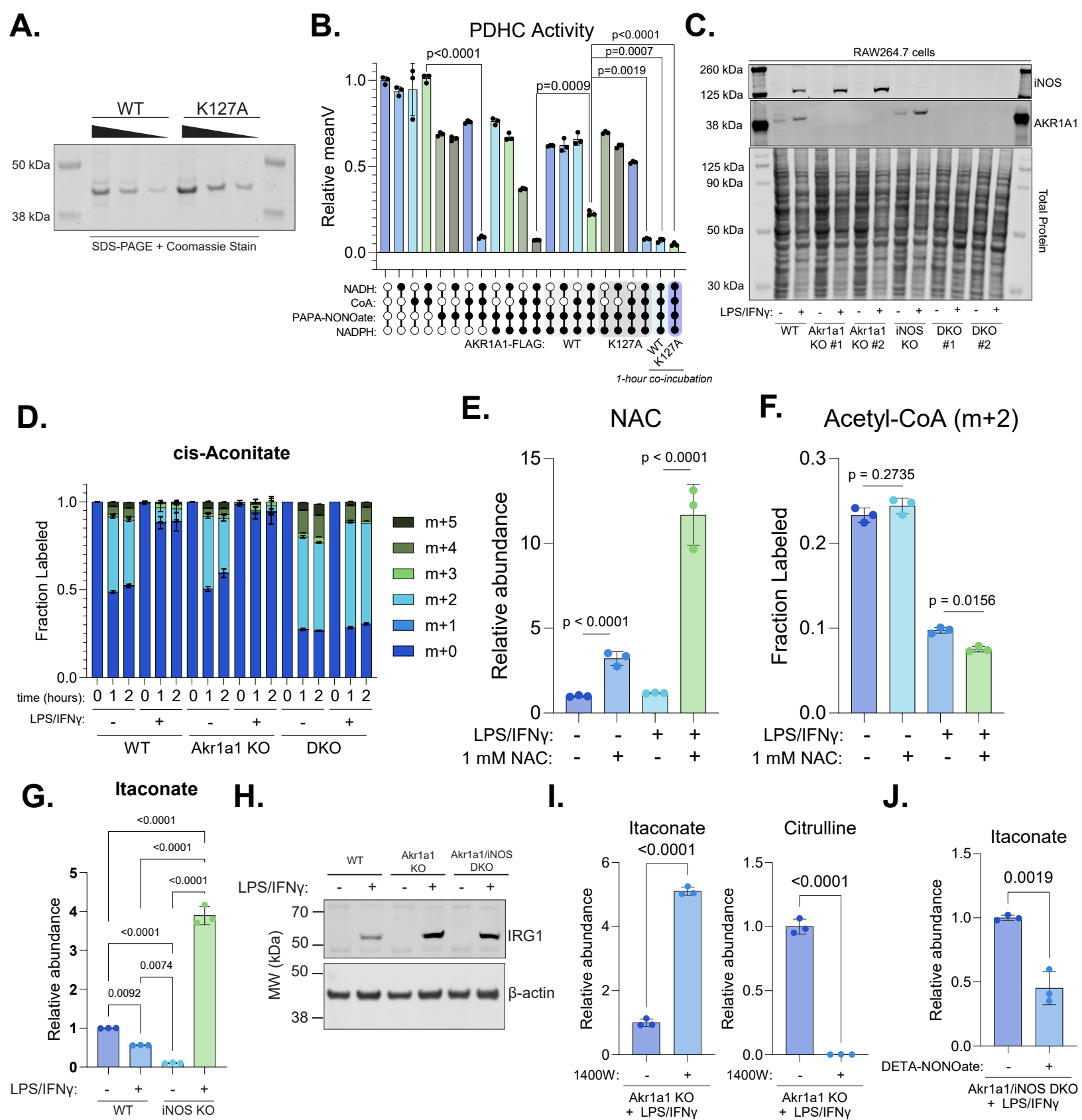

Supplement: Supplementary file 9 — Supplemental Figure 8AKR1A1 regulates TCA cycle by tempering the effect of NO• (A) Coomassie-stained SDS-PAGE gel showing purified recombinant AKR1A1-FLAG proteins. WT and catalytically inactive K127A mutant AKR1A1-FLAG were expressed in HEK293T cells and purified via anti-FLAG affinity chromatography. Serial dilutions (left to right) demonstrate purity. Expected molecular weight: ∼40 kDa. (B) Temporal requirement for Akr1a1 protection. Same conditions as (Fig. 5E) but with all conditions represented and two additional: Akr1a1-FLAG (WT or K127A) added after 2-h pre-incubation with NADH, NADPH, CoA, and PAPA-NONOate versus Akr1a1 added at time zero. Activity normalized to protein only control. Data represents mean ± SD, n = 3 independent reactions. (C) Immunoblot showing iNOS and AKR1A1 protein abundance for WT, Akr1a1 KO, iNOS KO, and Akr1a1/iNOS DKO clones (#1 and #2 indicate independent single clones) in RAW264.7 cells. All unstimulated or stimulated with LPS/IFNγ for 48-h. Total protein stain shown. (D) Isotopologue distribution of cis-Aconitate from conditions described in Fig. 5G. (E) Relative abundance of N-acetylcysteine (NAC) in RAW264.7 cells with or without LPS/IFNγ stimulation ± 1 mM NAC treatment for 48-h. Data represents mean ± SD, n = 3 biological replicates. (F) Fraction of 2-labeled acetyl-CoA from 2-h U–13C-d-glucose tracing in RAW264.7 cells unstimulated or stimulated with LPS/IFNγ ± 1 mM NAC treatment. Data represents mean ± SD, n = 3 biological replicates. (G) Relative abundance of itaconate in WT and iNOS KO RAW264.7 cells with or without 48-h LPS/IFNγ stimulation. Data represents mean ± SD, n = 3 biological replicates. (H) Immunoblot for IRG1 and beta-actin protein abundance for same conditions as Fig. 5H. (I) Relative abundance of itaconate (left) and citrulline (right) in Akr1a1 KO RAW264.7 cells with 48-h LPS/IFNγ with or without 200 μM 1400W. Data represents mean ± SD, n = 3 biological replicates. (J) Relative abundance of itaconate in Akr1 [file mmc8.pdf]

**A.**

Differential Gene Expression: Akr1a1 KO vs WT stimulated

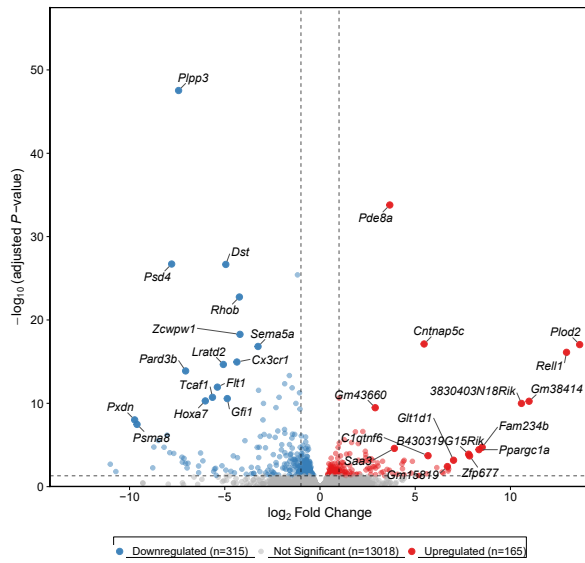

**B.**

IL-6

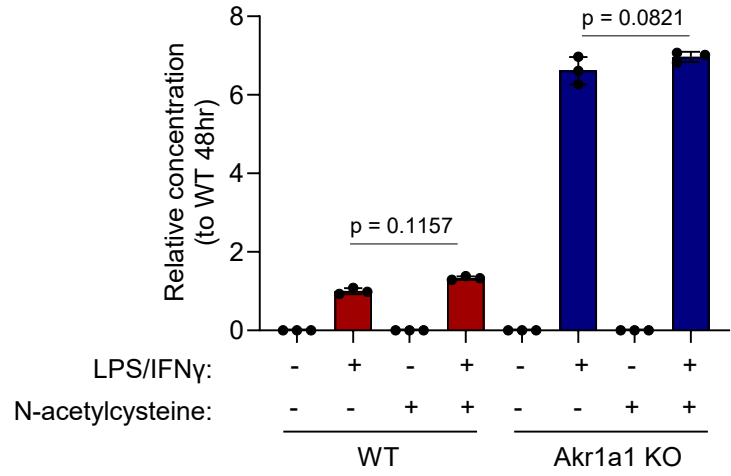

Supplement: Supplementary file 10 — Supplemental Figure 9Akr1a1-dependent transcriptome changes observed in stimulated RAW264.7 cells (A) Volcano plot showing differentially expressed genes in RAW264.7 cells comparing stimulated Akr1a1 KO versus WT (LPS/IFNγ, 48 h; n = 2 independent clonal replicates per genotype). Genes meeting significance threshold (p-adj <0.05) are colored by direction of change: upregulated in Akr1a1 KO (red), downregulated in Akr1a1 KO (blue), not significant (grey). Dashed lines indicate thresholds at p-adj = 0.05 (horizontal) and |log2FC| = 1 (vertical) for reference. Top 25 genes per direction (ranked by -log10(p-adj) × |log2FC|) are labeled. (B) IL-6 production by WT or Akr1a1 KO RAW264.7 cells with or without LPS/IFNγ stimulation ± 1 mM NAC treatment for 48-h. Data represents mean ± SD, n = 3 biological replicates. Statistical comparisons were performed using one-way ANOVA with Tukey's post hoc test for multiple comparisons. Exact p-values reported.Multimedia Component 9 [file mmc9.pdf]
