# Supplementary material for: Multi-omic analysis reveals nitric oxide dependent remodeling in classically activated macrophages and identifies negative regulation mediated by AKR1A1
Source: Redox Biol. 2026 Apr 22;93:104181. doi: 10.1016/j.redox.2026.104181 (PMC13137910; doi:10.1016/j.redox.2026.104181)
Supplement: Supplementary file 4 — Supplemental Figure 3iNOS-dependent transcriptome changes observed in stimulated RAW264.7 cells (A) Volcano plot showing differentially expressed genes in 48-h LPS/IFNγ stimulated iNOS knockout (iNOS KO) versus stimulated wildtype (WT) RAW264.7 cells (n = 3 biological replicates per genotype per condition). Genes meeting significance thresholds (p-adj <0.05 and |log2 fold change| > 1.0) are colored by direction of change: upregulated in iNOS KO (red), downregulated in iNOS KO (blue), not significant (grey). Dashed lines indicate thresholds at p-adj = 0.05 (horizontal) and |log2FC| = 1 (vertical). Top 25 genes per direction (ranked by -log10(p-adj) × |log2FC|) are labeled. (B–D) Lists of top 20 upregulated or downregulated protein/genes identified in each of the significance categories as described in Fig. 2A including both significant (concordant) (B), protein only (C), and RNA only (D), sorted by negative and positive log2FC of protein (B–C), or RNA (D).Multimedia Component 3 [file mmc3.pdf]

A.

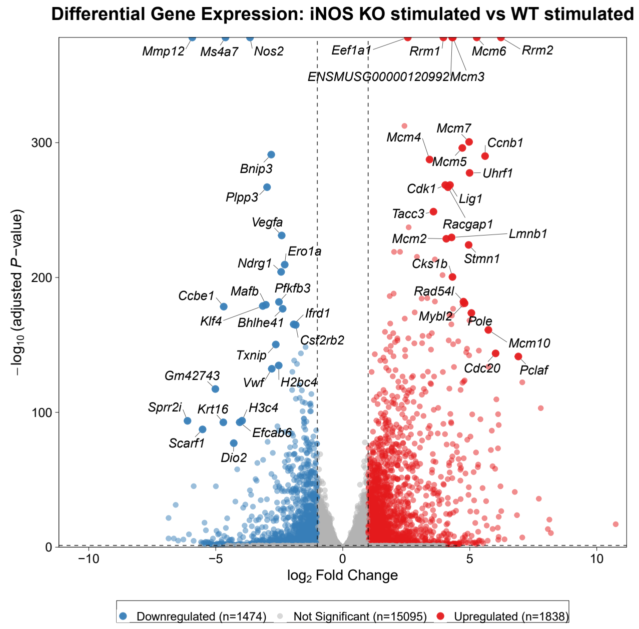

B.

Top 20 Protein/Genes: Both sig (concordant)

Protein	Negative Log2FC	Positive Log2FC
1	Nos2	Rad51ap1
2	Rsad2	Mak16
3	Bnip3	Incenp
4	Hmox1	Ect2
5	Plin2	Tpx2
6	Saraf	Bub1b
7	Cmpk2	Kif11
8	Ndst1	Ndc80
9	Serpinb9b	Racgap1
10	Slc37a2	Tex30
11	C3ar1	Fads2
12	Cd274	Fdx1
13	Rab7b	Irf8
14	Rap2a	Slc7a1
15	Peds1	Cep55
16	Cd86	Dlgap5
17	Tor4a	Birc5
18	Ggta1	Rrm2
19	Smpdl3a	Ccnb1
20	Akr1a1	Ndufv2

RNA	Negative Log2FC	Positive Log2FC
1	Nos2	Pbk
2	Bnip3	Pclaf
3	Txnip	Rrm2
4	Cox20	Kntc1
5	Pfkfb3	Plk1
6	Ndrg1	Kif4
7	Ero1a	Ndc80
8	Cd274	Kif15
9	Atp6v0d2	Ccnb1
10	Rsad2	Shcbp1
11	Map4k3	Knstrn
12	Rgs1	Spc24
13	Echdc3	Mcm6
14	Ckb	Kif11
15	Kdm3a	Dscc1
16	Naglu	Fanci
17	Gpnmb	Pole
18	Lyst	Uhrf1
19	Ndst1	Top2a
20	Slc37a2	Kif2c

C.

Top 20 Protein/Genes: Protein Only

Protein	Negative Log2FC	Positive Log2FC
1	Fabp7	Ndufa2
2	Atp2b3	Ndufs1
3	Ifit2	Sdhb
4	Usp18	Lias
5	Reep3	Rpf1
6	Psen1	Ndufa4
7	Ifit1	Ndufa10
8	Rap2c	Llph
9	Tspyl1	Sdha
10	Slc28a2	Nrp1
11	Maob	Ndufs3
12	Gpat3	Gng10
13	Ada	Irf2
14	Gvin1	Hic2
15	Ggh	Sdhc
16	Ldah	Ndufa6
17	Tlr3	Abce1
18	Atp8a1	Dgkd
19	Atxn7	Nsun5
20	Abcb6	Pold3

D.

Top 20 Protein/Genes: RNA Only

RNA	Negative Log2FC	Positive Log2FC
1	Acad12	Cit
2	Mtss1	Nup210
3	Pdk2	Fam83g
4	Arhgap22	Dnph1
5	Eno2	Orc1
6	Naga	Dck
7	Vps37b	Topbp1
8	Rpgr	Nup43
9	Eif4a2	Ptma
10	Dnm1	Nes
11	Pacc1	Pcyox1l
12	Bsg	Dkc1
13	Tmem115	Nup133
14	Smg9	Apex1
15	Ptrh1	Sdf2l1
16	Tnfrsf26	Rps16
17	Pacs2	Gm7324
18	Cpeb4	Rps6
19	Slc2a1	Snrpf
20	Stam	Isyna1
